# Exploring the Feasibility of 16S rRNA Short Amplicon Sequencing-Based Microbiota Analysis for Microbiological Safety Assessment of Raw Oyster

**DOI:** 10.4014/jmb.2302.02007

**Published:** 2023-06-09

**Authors:** Jaeeun Kim, Byoung Sik Kim

**Affiliations:** Department of Food Science and Biotechnology, ELTEC College of Engineering, Ewha Womans University, Seoul 03760, Republic of Korea

**Keywords:** Microbiota profiling, V3-V4 amplicon sequencing, food safety, microbiological safety assessment, *Vibrio parahaemolyticus*

## Abstract

16S rRNA short amplicon sequencing-based microbiota profiling has been thought of and suggested as a feasible method to assess food safety. However, even if a comprehensive microbial information can be obtained by microbiota profiling, it would not be necessarily sufficient for all circumstances. To prove this, the feasibility of the most widely used V3-V4 amplicon sequencing method for food safety assessment was examined here. We designed a pathogen (*Vibrio parahaemolyticus*) contamination and/or *V. parahaemolyticus*-specific phage treatment model of raw oysters under improper storage temperature and monitored their microbial structure changes. The samples stored at refrigerator temperature (negative control, NC) and those that were stored at room temperature without any treatment (no treatment, NT) were included as control groups. The profiling results revealed that no statistical difference exists between the NT group and the pathogen spiked- and/or phage treated-groups even when the bacterial composition was compared at the possible lowest-rank taxa, family/genus level. In the beta-diversity analysis, all the samples except the NC group formed one distinct cluster. Notably, the samples with pathogen and/or phage addition did not form each cluster even though the enumerated number of *V. parahaemolyticus* in those samples were extremely different. These discrepant results indicate that the feasibility of 16S rRNA short amplicon sequencing should not be overgeneralized in microbiological safety assessment of food samples, such as raw oyster.

## Introduction

During the past two decades, the 16S rRNA short amplicon sequencing method has been widely used to investigate bacterial communities in various environments, together with the advent of deep sequencing (also known as next-generation sequencing) technologies [1-4]. This method targets the bacterial 16S rRNA gene, which consists of nine hypervariable regions interspaced and flanked by conserved sequences among different bacterial species [5]. By amplifying and sequencing these species-specific hypervariable loci using universal PCR primers (which anneal to the conserved sequences), researchers can identify the bacterial composition of given samples [6]. Among many hypervariable regions, the V3-V4 region (variable region 3 to variable region 4) and other short variable regions (such as V4) have been frequently examined, because they provide a basic insight into bacterial composition at a reasonable cost. Technical limitation of the dominantly utilized platform, Illumina, also confines the amplification range to be short: the Illumina devices usually allows approximately 500 nucleotides per joined paired-end read.

Although 16S rRNA short amplicon sequencing could provide tremendous data, practical problems also exist. In general, amplicon sequencing for the short variable regions of 16S rRNA gene can give the genus level information at best, but more detailed analysis on the species level is mostly impossible [7]. Recently, the amplicon sequence variant (ASV) analysis has been introduced but this method still relies on the long-enough amplicon, and importantly, has limitation in identifying detailed taxa belonging to the certain genus, like *Bacillus* and *Vibrio*, using short amplicon sequence data [8, 9]. Accordingly, the 16S rRNA short amplicon sequencing is mainly employed to compare the ecological composition of bacterial populations in different environments or niches showing the genus level differences at least, and to infer their consequent characteristic changes. In case of food science, bacterial composition can influence various metabolites, which govern flavor and taste of food, and thus such microbial profiling method has been successfully adapted for food quality assessments [10, 11].

Nonetheless, not a few studies have discussed food safety level through this technique by emphasizing the dominance of certain family or genus that includes potentially hazardous foodborne pathogens. For example, Shehata *et al*. [12] and Artimová *et al*. [13] employed the V3-V4 or V4 amplicon sequencing to explore microbiota of commercially available finfish fillets or fresh-consumed plant products, respectively, and assess the potential risk of food poisoning. Notably, however, no such study has actually examined feasibility of 16S rRNA short amplicon sequencing for food safety assessment by directly comparing the conventional enumeration method and the microbiota profiling analysis after artificial spiking of foodborne pathogens [14-16].

In this study, we designed a *Vibrio parahaemolyticus* contamination and improper storage model of raw oysters and monitored changes in the microbiota to evaluate the feasibility of 16S rRNA short amplicon sequencing-based microbiota analysis for food safety assessment. Actual number of the spiked *V. parahaemolyticus* cells and the microbial composition characteristics of various experimental groups (pathogen bloomed and not bloomed groups) were examined. The results from this study showing the discrepancy between conventional enumeration method and short amplicon sequencing-based methods does provide insights into the appropriateness and limitations of the 16S rRNA short amplicon sequencing-based microbiota profiling for microbiological safety assessment of food.

## Materials and Methods

### Sample Preparation

Shucked raw oysters (*Crassostrea gigas*) packed in polyethylene bags filled with water were obtained from a retail market on-site in Seoul, South Korea. The provided water (200 ml) from each package of oysters was collected and mixed in a sterilized beaker. Oysters were then placed into the beaker, which was stored in a refrigerator (4°C) to retain homogeneity among the samples. After 1 h of adaptation, the samples (20 ml of the mixed water and one oyster per sample) were transferred into sterilized 50 ml conical tubes. The average weight of the oysters was 12.4 ± 2.2 g. All the procedures were conducted in a biosafety cabinet Class IIa with sterilized tongs to avoid external contamination.

### Bacterial Strain and Bacteriophage

For the enumeration of the artificially contaminated *V. parahaemolyticus*, a spontaneous mutant that was resistant to rifampicin was selected, as follows. An overnight culture of *V. parahaemolyticus* FORC_023 strain was transferred daily to fresh Luria-Bertani (LB) medium supplemented with 2% (w/v) NaCl (LBS) and increasing amounts of rifampicin (from 2.5 to 100 μg/ml). The final transferred and overnight-incubated culture was then spread on LBS agar plates containing rifampicin (100 μg/ml) for selection of a single colony. The resulting colony was confirmed as rifampicin-resistant *V. parahaemolyticus* and renamed FORC_023_Rif^R^. No growth difference between the parental FORC_023 strain and the FORC_023_Rif^R^ strain was observed in vitro. Meanwhile, the *V. parahaemolyticus*-specific bacteriophage VPT02 was amplified and stored in sodium chloride–magnesium sulfate (SM) buffer, as described previously [17, 18]. Notably, this lytic bacteriophage can infect a wide range of *V. parahaemolyticus* strains, among which FORC_023 is one of the most susceptible.

### Artificial Contamination and Phage Treatment of the Oysters

To profile the microbial structures of oysters upon the pathogen contamination and blooming, the oysters were artificially spiked with *V. parahaemolyticus* FORC_023_Rif^R^, in the absence or presence of phage VPT02, and stored at 25°C. The phage was added to mimic the colonization failure of contaminating pathogen on food. Specifically, 10 ml of FORC_023_Rif^R^ culture [grown at LBS to the optical density at 600 nm (OD_600_) of 1.0] was washed three times and diluted with phosphate-buffered saline (PBS) to OD_600_ of 0.5, which corresponded to 1 × 10^7^ CFU/ml. Considering the previously reported hazardous contamination level of *V. parahaemolyticus* in oysters [19], approximately 100 CFU/g oyster of the washed FORC_023_Rif^R^ strain was added to each conical tube containing the adapted oyster. For the phage treatment groups, VPT02 was added to the samples at a multiplicity of infection (MOI) of 1,000. Accordingly, samples of *Vibrio* treatment only group (VO), *Vibrio* and phage treatment group (VP), and phage treatment only group (PO) were prepared. For the negative control (NC) and no treatment (NT) groups, PBS and SM buffer were added instead of the pathogen and phage, respectively. All the samples except the NC were then stored at 25°C for 6 h. After incubation, the contents of each conical tube were poured into a filter bag with sterile buffered peptone water (BPW; 9 ml/g oysters), and the microbial content was detached from the oysters using the Pulsifier II (Microgen Bioproducts, UK) for 15 sec. One ml of the resulting solution was serially diluted with BPW, and 100 μl of diluent was spread on LBS agar plates containing rifampicin (100 μg/ml) for the enumeration of *V. parahaemolyticus* FORC_023_Rif^R^. Bacterial cells from the remaining sample solution were collected by centrifugation (15 min at 7,871 ×*g*) and stored at -80°C for metagenomic DNA extraction.

### Metagenomic DNA Extraction

The total metagenomic DNA of each sample was extracted using a DNeasy PowerSoil Pro Kit (Qiagen, Germany), according to the manufacturer’s instructions. The purity and concentration of the resulting DNA were measured using a NanoQuant plate and Spark microplate reader (Tecan, Switzerland). Prior to subsequent analysis, the DNA samples were kept at -20°C.

### Deep Sequencing and Preprocessing

16S rRNA amplicon sequencing was conducted at Sanigen Co. Ltd. (Korea). Forward (5’-TCGTCGGCAGCGTCAGATGTGTATAAGAGACAGCCTACGGGNGGCWGCAG-3’) and reverse (5’-GTCTCGTGGGCTCGG AGATGTGTATAAGAGACAGGACTACHVGGGTATCTAATCC-3’) primers were used to amplify the V3-V4 region of the bacterial 16S rRNA genes from the extracted DNAs. The PrimeSTAR HS DNA polymerase (Takara, Japan) was used in PCR amplification, according to the following conditions: initial denaturation (95°C, 3 min), 25 cycles of amplification [denaturation (95°C, 30 sec), annealing (55°C, 30 sec), and elongation (72°C, 30 sec)], and final elongation (72°C, 3 min). The resulting PCR product was purified using Agencourt AMPure XP beads (Beckman Coulter, USA) according to the manufacturer’s instructions. An Illumina Nextera XT index kit (Illumina, USA) was used to construct the indexed library for deep sequencing. After validating the size, quality, and quantity of the constructed library using a Bioanalyzer 2100 (Agilent, USA) and Qubit4.0 (Thermo Fisher Scientific, USA), deep sequencing was conducted via the Illumina MiSeq platform (Illumina) with a paired-end (2 × 300 bp) sequencing mode. For preprocessing of the raw sequencing reads, artificial sequences such as the barcode index and primer sequences, as well as low-quality scored sequences (Q < 30), were trimmed out using Trimmomatic (ver. 0.39) [20]. Chimeric sequences were removed using the DADA2 pipeline via QIIME2 (ver. 2021.11) [21].

### Microbiota Profiling and Diversity Analysis

The operational taxonomic unit (OTU) sequence classification was performed using the SILVA reference database (release 138) [22]. The taxonomic assignment, diversity analyses and result visualization were conducted using QIIME2 (ver. 2022.2). Rare fraction curves of Shannon index and microbial alpha diversity of Shannon and Faith’s phylogenetic diversity (PD) indices were calculated using QIIME2. Beta-diversity was analyzed using weighted UniFrac distance metrics utilizing QIIME2.

### Statistical Analysis

Statistical analysis was conducted as indicated in each figure legend using GraphPad Prism software (ver. 8.4.3; USA).

## Results

### *V. parahaemolyticus* Population Changes Following Artificial Treatments

**Fig. 1:**
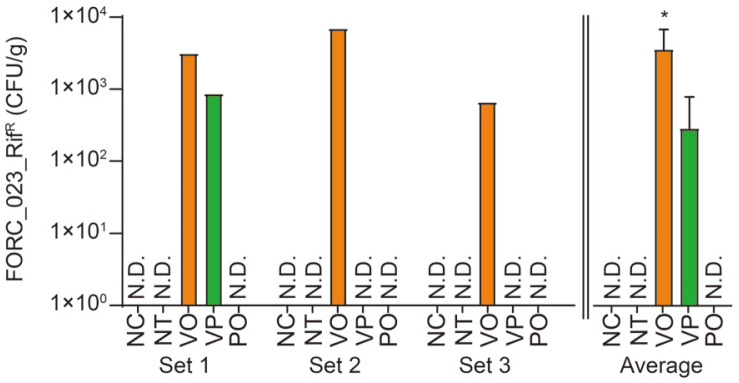
Enumeration results of the spiked *V. parahaemolyticus* FORC_023_Rif^R^ in the homogenized sample solution. N.D. (No detection) indicates that the abundance of *V. parahaemolyticus* was lower than the detection limit. Means and standard deviations are presented on a right graph. The statistical difference was calculated using the Kruskal-Wallis test. *, *p* < 0.05; NC, negative control; NT, no treatment group; VO, *Vibrio* treatment only group; VP, *Vibrio* and phage treatment group; PO, phage treatment only group.

Before the metagenome sampling, we examined whether our experimental conditions indeed mimic improper storage situations for food poisoning and thus affect the safety level of oysters by directly enumerating the number of *V. parahaemolyticus* cells in the samples. Notably, no rifampicin-resistant bacterial cells were present in the initial oysters (data not shown). However, following the artificial spiking and incubation, the number of *V. parahaemolyticus* FORC_023_Rif^R^ increased to approximately 3.6 × 10^3^ CFU/g (Fig. 1, *Vibrio* Only, VO). If the bacteriophage VPT02 was added after the pathogen spiking, the number of bloomed *V. parahaemolyticus* populations was significantly reduced compared with the VO group (Fig. 1, *Vibrio* + Phage, VP). Specifically, two out of three VP samples contained less than 1 × 10^2^ CFU/g (detection limit of the experiment) of *V. parahaemolyticus* FORC_023_Rif^R^, and one sample exhibited a substantial reduction (from 3.1 × 10^3^ CFU/g to 8.6 × 10^2^ CFU/g). As expected, no *V. parahaemolyticus* FORC_023_Rif^R^ were recovered from the NC, NT and Phage Only (PO) groups. These results confirmed that the artificial spiking and subsequent incubation accurately mimicked the most important safety issue of raw oysters.

### Taxonomic Signatures of Improperly Stored Raw Oysters

**Fig. 2:**
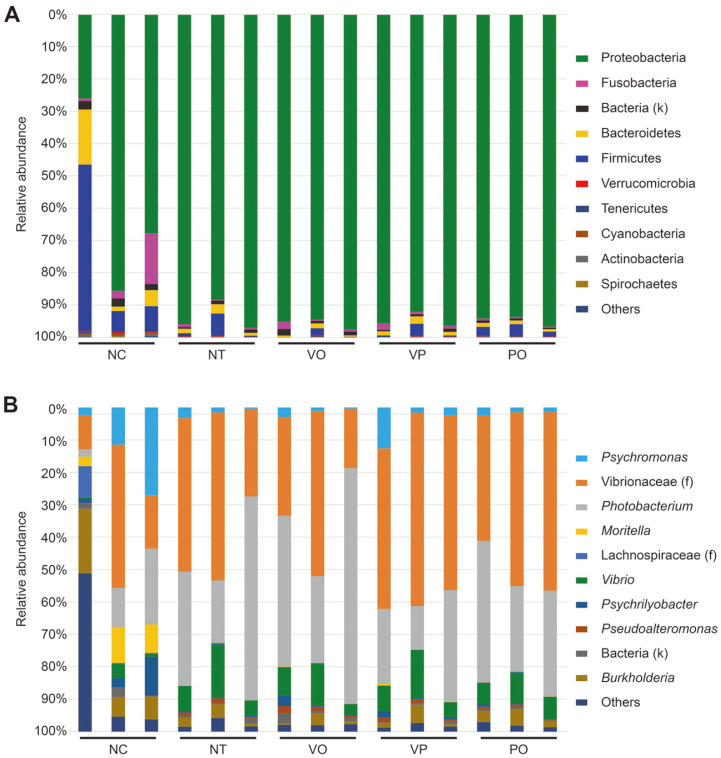
Microbial composition of each sample at the (A) phylum and (B) the possible lowest-rank taxa, family/genus level. The phyla and genera observed to be greater than 0.5% in at least one sample are presented. The remaining phyla and genera are indicated as others. The *Vibrio*naceae family shown in panel (B) does not include the *Vibrio* genus.

The bacterial composition in the raw oyster samples after artificial spiking with *V. parahaemolyticus* and/or phage treatment was analyzed taxonomically at the phylum and the possible lowest-rank taxa, family/genus levels. As shown in Fig. 2A, regardless of which treatment was applied to the raw oysters, all the samples (except those belonging to the NC group) were largely dominated by Proteobacteria at the phylum level. The relative abundance of Proteobacteria was 93.8 ± 4.8% in the NT group, 95.8 ± 1.6% in the VO group, 94.8 ± 2.3% in the VP group, and 94.9 ± 1.6% in the PO group. For the NC group, Proteobacteria was also the most abundant phylum but was significantly less abundant than in the other groups (59.9 ± 30.6%) and was followed by Firmicutes (21.9 ± 25.6%) and Bacteroidetes (7.8 ± 8.2%).

At the possible lowest-rank taxa, family/genus level, all the samples except the NC group exhibited similar bacterial profiles, which is reminiscent of the results from the phylum level analysis (Fig. 2B). In general, the NT, VO, VP, and PO groups were primarily composed of members of the *Vibrio*naceae family (33.3% to 54.4%) and Photobacterium (23.6% to 48.7%). Compared to these groups, the NC group samples exhibited a more diverse bacterial composition at the genus level. In fact, three oyster samples in the NC group showed distinct bacterial ecological characteristics with each other: the genus Burkholderia (19.9%) and the members of *Vibrio*naceae family (44.2%) predominated in the bacterial population of the 1^st^ and 2^nd^ samples, respectively, while no single genus obviously predominated in the 3^rd^ sample of NC group (27.1% for *Psychromonas*, 23.4% for *Photobacterium*, and 16.5% for the member of Vibrionaceae family). Notably, all the NC group samples contain more abundant minor genera than the other group samples, as shown as ‘Others’ in Fig. 2B. In case of the 1^st^ sample of NC group, about 48.9% of the total bacteria belong to these minor genera. These results indicate that the raw oysters may have distinct initial bacterial compositions and neither of presence nor absence of *V. parahaemolyticus* induces their own characteristic bacterial composition changes at the possible lowest-rank taxa, family/genus level even after the improper storage.

### Comparison of Bacterial Diversity Indices

**Fig. 3:**
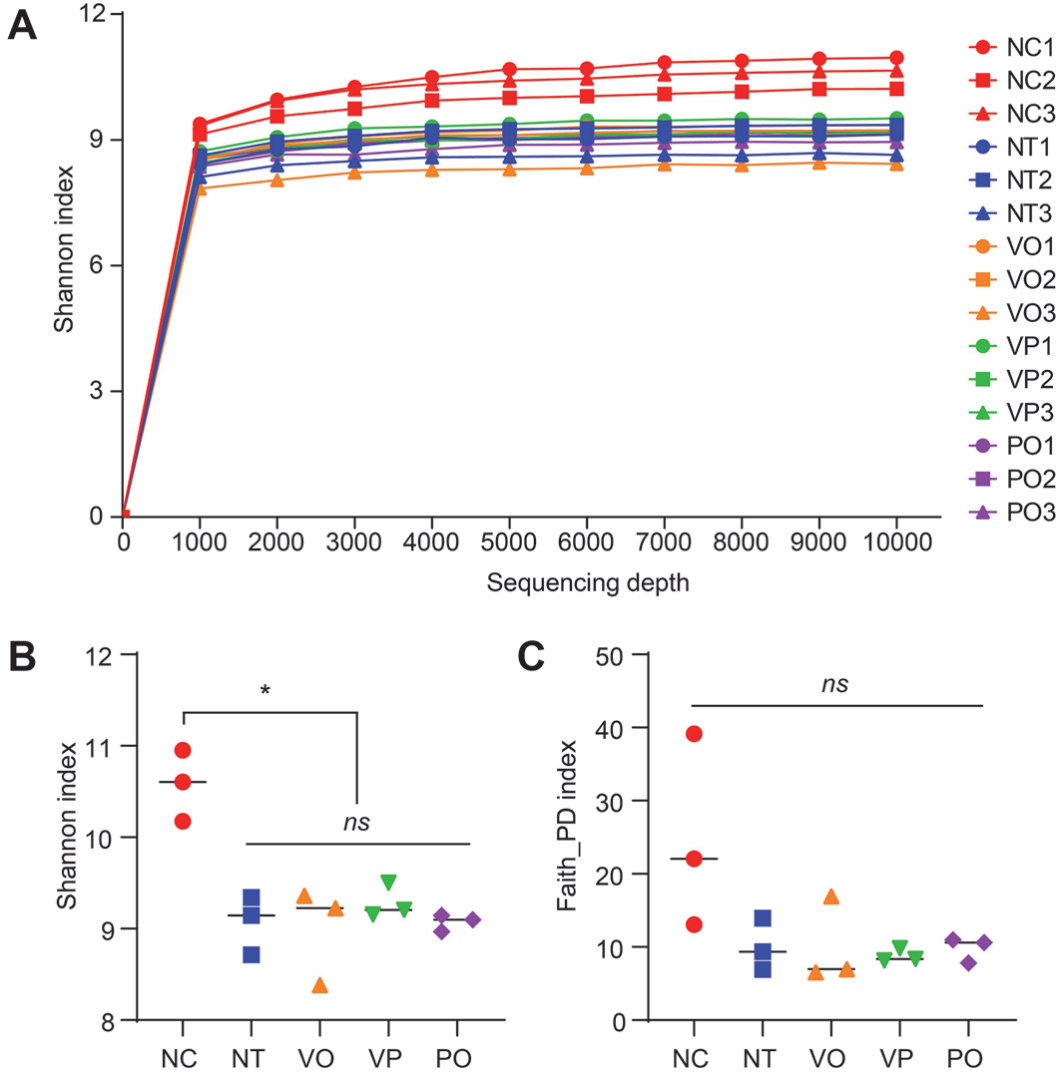
Results of the alpha-diversity analysis. (**A**) Shifts in the Shannon index of each sample depending on the sequencing depth. Comparison of the (**B**) Shannon diversity and (**C**) Faith’s PD indices of each group. The vertical lines represent the median of each group. The statistical differences were calculated using the Kruskal-Wallis test. *, *p* < 0.05; ns, non-significant.

To compare the bacterial diversity among the groups more quantitatively, the number of sequencing reads of each sample (read depth per sample) was normalized to 9,000 via rarefaction (Table 1). Notably, this depth was enough for the saturation of bacterial diversity in each sample (Fig. 3A). For the Shannon index, which reflects both the species richness and evenness of a given microbial ecosystem, the NC group exhibited a statistical difference compared with all the other groups (Kruskal-Wallis test, *p* = 0.0427) whereas no difference was observed among the NT, VO, VP, and PO groups (Fig. 3B). However, in the case of Faith’s phylogenetic diversity (PD) index, which measures the species richness based on the phylogenetic relationship, no statistical difference was observed among all groups (Kruskal-Wallis test, *p* = 0.2369) (Fig. 3C). These results suggest that the diversity reduction observed in the NT, VO, VP, and PO groups might be caused by the expansion of certain bacterial taxa, which also exists in the NC group or is phylogenetically close with the taxa in the NC group.

### Bacterial Composition Clustering

**Fig. 4:**
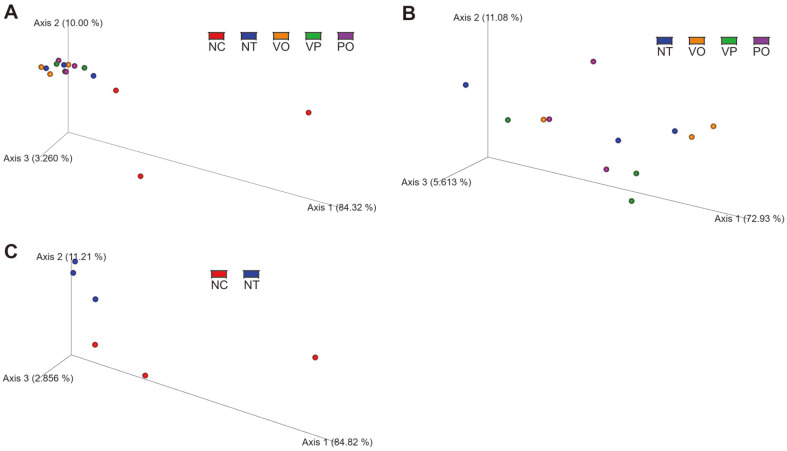
Beta-diversity PCoA plots based on weighted UniFrac. (**A**) Total samples. (**B**) The NT, VO, VP and PO group samples. (**C**) The NC and NT group samples. Each dot indicates a single sample.

Lastly, the bacterial composition of the raw oyster samples was analyzed via three-dimensional Principal Coordinates Analysis (3D-PCoA), based on the weighted UniFrac distance. Consistent with the taxonomic analysis results (Figs. 2A and 2B), all the samples, except those in the NC group, formed a single distinct cluster (Fig. 4A). Each sample of the NC group had largely separated each other, as this can be deduced from the possible lowest-rank taxa, family/genus level analysis (Fig. 2B). Notably, other groups, namely the NT, VO, VP, and PO groups, did not establish its own cluster even if the same 3D-PCoA was conducted without the NC group (Fig. 4B). These results indicate that the bacterial compositions of the NT, VO, VP, and PO groups were indistinctive.

## Discussion 

Recently, many studies have explored microbial composition of food using the 16S rRNA short amplicon sequencing method. It is a powerful tool to demonstrate the indigenous microbial community and shift of the microbiota profile during condition changes, which occur while the processing, storage, and distribution of food. With general recognition by food microbiologists on inherent limitation of the technique, the 16S rRNA short amplicon sequencing was widely used for food quality assessments. In the meantime, however, not a few studies have also argued that the microbiota analysis can be used to assess the safety level of given food items, providing empirical information to establish control points in food processing steps. These claims were based on the observed expansion of certain bacterial family and/or genus in relative abundance. For example, Kim *et al*. [23] demonstrated that the relative abundance of family Enterobacteriaceae, which includes major foodborne pathogens such as *Escherichia* spp. and *Shigella* spp., was significantly increased during the seed soaking step of alfalfa sprout production and, consequently, suggested the addition of further interventions during this step. A separate study by Kim *et al*. [15] demonstrated that the proportion of potential pathogens in the microbiota of sea cucumbers is higher in November than in August and argued that the ingestion of sea cucumbers poses a risk in November, even if the seawater temperature is relatively low. However, studies that examined the feasibility of 16S rRNA short amplicon sequencing-based microbiota analysis for food safety assessment by employing actual pathogen contamination at a threatening level were limited.

In this study, we designed a *V. parahaemolyticus* contamination and improper storage model for raw oysters and adapted both the conventional enumeration method and 16S rRNA short amplicon sequencing-based microbiota profiling analysis. The direct enumeration for experimental samples indicated a sufficient degree of contamination of the *V. parahaemolyticus* FORC_023_Rif^R^ for food poisoning (Fig. 1) [19]. The effect of phage VPT02 that prevents colonization of the pathogen was also noticeable. Using this conventional method, we demonstrated that the artificial spiking of *V. parahaemolyticus* and phage treatment suitably represent foodborne pathogen contamination and prevention situations in seafood.

**Fig. 5:**
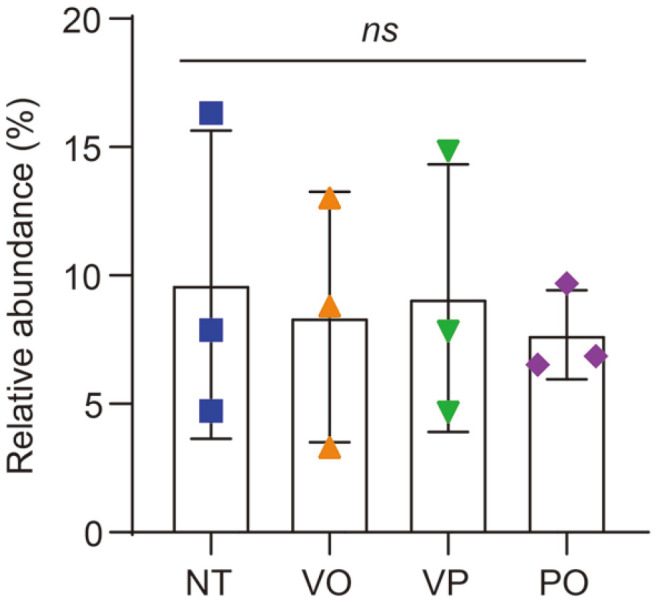
Relative abundance of the *Vibrio* genus for groups with different treatments. The data are presented as the means ± standard deviations from three samples. The statistical differences were calculated using one-way ANOVA. *ns*, nonsignificant.

Regarding the microbiota profiling, the bacterial composition as well as the alpha- and beta-diversity indices of the NC group were distinct from those of the other groups. However, no significant differences were observed among the NT, VO, VP, and PO groups, indicating that the 16S rRNA short amplicon sequencing-based microbiota profiling method could not distinguish the pathogen containing, and thus potentially hazardous food from non-hazardous food (Figs. 2-4). This suggests that the contamination and blooming-up of the pathogen is not a governing factor for the microbiota changes in oysters during improper storage. The main difference between the NC group and the other groups was rather the storage temperature. Consistent with this, the 3D-PCoA of the samples in the NC and NT groups, which differed only regarding the storage temperature, resulted in a separated clustering (Fig. 4C). Most importantly, when combined with the enumeration results, these results indicate that the microbiota profiling method we adapted (*i.e.*, V3-V4 amplicon sequencing) is an unsuitable method to predict and/or assess the pathogen contamination status of raw oysters. In contrast to the previous studies [12, 13, 15, 23], no correlation was observed between the presence of foodborne pathogen (*V. parahaemolyticus*) and the relative abundance of potentially hazardous genus (*Vibrio*) (Figs. 2B and 5).

It is important to understand the possible reasons for this discrepancy between the results of the conventional assessment method (enumeration) and the microbiota profiling method. First, the resolution of V3-V4 amplicon sequencing might not be sufficient to capture the blooming pathogen *V. parahaemolyticus*. In general, the choice of amplification region in the 16S rRNA gene greatly affects the bacterial community metabarcoding results [24]. For example, one can obtain a higher resolution for lower-rank taxa (even at the species level) if the longer region of the 16S rRNA gene is sequenced [25]. In this study, the lowest-rank taxa we could assign was the genus *Vibrio*, and no specific OTU was matched to *V. parahaemolyticus* (data not shown). Similarly, the previous studies failed to assign any specific foodborne pathogens from their short amplicon sequencing-based metabarcoding analyses [12, 13, 23].

Second, the inherent microbiota of the raw oysters might disguise the contamination-mediated changes in the bacterial profile. It is known that various *Vibrio* species, such as *V. campbellii*, *V. rotiferianus*, and *V. owensii*, are commonly present in oysters [26]. Since these inherent *Vibrio* species could also bloom during the improper storage of raw oysters, the relative abundance of the genus *Vibrio* in the NT, VO, VP, and PO groups might do not exhibit any statistical differences even if the VO and VP samples were spiked with *V. parahaemolyticus* (Fig. 5). If the taxa that are evolutionally associated with the pathogens under examination rarely pre-exist in a food sample, the contamination and/or dominance of such pathogens could be assessed, even though the increase in relative abundance at the genus or family level. In contrast, if food microbiota contained the evolutionally related taxa with concerning pathogens, the feasibility of the 16S rRNA short amplicon sequencing-based microbiota profiling would be significantly limited as clearly evidenced by this study.

In conclusion, we evaluated the feasibility of V3-V4 amplicon sequencing-based microbiota analysis for food safety assessment using an improper storage model of raw oysters. Although this method has provided valuable insights into the microbial ecological characteristics and quality of various foods [10, 11, 14, 15, 27], our results demonstrated that the following points must be considered when microbiota profiling is applied for food safety assessment: 1) use sequencing methods that can provide sufficiently high resolution for the taxonomic classification of pathogens being examined, and 2) the results can be significantly influenced by the basal-level contents of the inherent microbiota of the given food. Regarding the first point, the recently suggested sequencing and analysis methods should be considered in the food safety field [28-30]. Regarding the second point, the safety of raw food and unprocessed food, such as raw seafood and green produce, should not be inferred or judged simply based on the results of 16S rRNA short amplicon sequencing. Metagenomic and/or metatranscriptomic approaches would help overcome these limitations of the microbiota profiling [31].

## Data Availability

The raw sequence data generated in this study were deposited into the Sequence Read Archive at the National Center for Biotechnology Information under the accession number PRJNA885990.

## Figures and Tables

**Table 1:** Read numbers and alpha diversity indices of each sample.

	Raw read numbers	Denoised read number	Normalized reads	Observed OTU	Shannon index
NC1	131,577	107,595	9,000	768	8.95
NC2	114,093	93,737	9,000	238	6.61
NC3	139,026	114,425	9,000	246	6.82
NT1	122,763	104,746	9,000	159	5.57
NT2	142,859	121,421	9,000	262	6.08
NT3	131,510	112,347	9,000	114	4.95
VO1	140,222	118,257	9,000	128	5.51
VO2	136,319	112,403	9,000	180	5.70
VO3	142,382	120,773	9,000	105	4.68
VP1	133,618	113,597	9,000	156	5.78
VP2	128,888	106,884	9,000	181	5.70
VP3	164,996	140,363	9,000	142	5.48
PO1	131,650	110,020	9,000	186	5.66
PO2	140,772	119,419	9,000	176	5.66
PO3	139,671	119,223	9,000	139	5.38

## References

[1] Dethlefsen L, Huse S, Sogin ML, Relman DA (2008). The pervasive effects of an antibiotic on the human gut microbiota, as revealed by deep 16S rRNA sequencing. PLoS Biol..

[2] Janssen PH (2006). Identifying the dominant soil bacterial taxa in libraries of 16S rRNA and 16S rRNA genes. Appl. Environ. Microbiol..

[3] Kim M, Chun J (2005). Bacterial community structure in kimchi, a Korean fermented vegetable food, as revealed by 16S rRNA gene analysis. Int. J. Food Microbiol..

[4] Revetta RP, Pemberton A, Lamendella R, Iker B, Santo Domingo JW (2010). Identification of bacterial populations in drinking water using 16S rRNA-based sequence analyses. Water Res..

[5] Neefs JM, Van de Peer Y, De Rijk P, Chapelle S, De Wachter R (1993). Compilation of small ribosomal subunit RNA structures. Nucleic Acids Res..

[6] Lane DJ, Pace B, Olsen GJ, Stahl DA, Sogin ML, Pace NR (1985). Rapid determination of 16S rRNA sequences for phylogenetic analyses. Proc. Natl. Acad. Sci..

[7] Martínez-Porchas M, Villalpando-Canchola E, Vargas-Albores F (2016). Significant loss of sensitivity and specificity in the taxonomic classification occurs when short 16S rRNA gene sequences are used. Heliyon.

[8] Hakovirta JR, Prezioso S, Hodge D, Pillai SP, Weigel LM (2016). Identification and analysis of informative single nucleotide polymorphisms in 16S rRNA gene sequences of the *Bacillus cereus* group. J. Clin. Microbiol..

[9] King WL, Siboni N, Kahlke T, Green TJ, Labbate M, Seymour JR (2019). A new high throughput sequencing assay for characterizing the diversity of natural *Vibrio* communities and its application to a pacific oyster mortality event. Front. Microbiol..

[10] Bassey AP, Chen Y, Zhu Z, Odeyemi OA, Frimpong EB, Ye K (2021). Assessment of quality characteristics and bacterial community of modified atmosphere packaged chilled pork loins using 16S rRNA amplicon sequencing analysis. Food Res. Int..

[11] de Boer P, Caspers M, Sanders J-W, Kemperman R, Wijman J, Lommerse G (2015). Amplicon sequencing for the quantification of spoilage microbiota in complex foods including bacterial spores. Microbiome.

[12] Shehata HR, Mitterboeck TF, Hanner R (2020). Characterization of the microbiota of commercially traded finfish fillets. Food Res. Int..

[13] Artimová R, Játiová M, Baumgartnerová J, Lipková N, Petrová J, Maková J (2023). Microbial communities on samples of commercially available fresh-consumed leafy vegetables and small berries. Horticulturae.

[14] Choi H, Hwang BK, Kim B-S, Choi SH (2020). Influence of pathogen contamination on beef microbiota under different storage temperatures. Food Res. Int..

[15] Kim TY, Lee JJ, Kim BS, Choi SH (2017). Whole-body microbiota of sea cucumber (*Apostichopus japonicus*) from South Korea for improved seafood management. J. Microbiol. Biotechnol..

[16] Lee JJ, Kim TY, Choi SH, Kim BS (2017). Analysis of the bacterial microbiome in the small octopus, *Octopus variabilis*, from South Korea to detect the potential risk of foodborne illness and to improve product management. Food Res. Int..

[17] You HJ, Lee JH, Oh M, Hong SY, Kim D, Noh J (2021). Tackling *Vibrio parahaemolyticus* in ready-to-eat raw fish flesh slices using lytic phage VPT02 isolated from market oyster. Food Res. Int..

[18] Lee JH, Oh M, Kim B-S (2023). Phage biocontrol of zoonotic food-borne pathogen *Vibrio parahaemolyticus* for seafood safety. Food Control.

[19] Su YC, Liu C (2007). *Vibrio parahaemolyticus*: a concern of seafood safety. Food Microbiol..

[20] Bolger AM, Lohse M, Usadel B (2014). Trimmomatic: a flexible trimmer for Illumina sequence data. Bioinformatics.

[21] Callahan BJ, McMurdie PJ, Rosen MJ, Han AW, Johnson AJA, Holmes SP (2016). DADA2: High-resolution sample inference from Illumina amplicon data. Nat. Methods.

[22] Pruesse E, Quast C, Knittel K, Fuchs BM, Ludwig W, Peplies J (2007). SILVA: a comprehensive online resource for quality checked and aligned ribosomal RNA sequence data compatible with ARB. Nucleic Acids Res..

[23] Kim SY, Ban GH, Hong YW, Jang MJ, Kim SA (2022). Microbiome shifts in sprouts (alfalfa, radish, and rapeseed) during production from seed to sprout using 16S rRNA microbiome sequencing. Food Res. Int..

[24] Bukin YS, Galachyants YP, Morozov I, Bukin S, Zakharenko A, Zemskaya T (2019). The effect of 16S rRNA region choice on bacterial community metabarcoding results. Sci. Data.

[25] Johnson JS, Spakowicz DJ, Hong BY, Petersen LM, Demkowicz P, Chen L (2019). Evaluation of 16S rRNA gene sequencing for species and strain-level microbiome analysis. Nat. Commun..

[26] King WL, Kaestli M, Siboni N, Padovan A, Christian K, Mills D (2021). Pearl oyster bacterial community structure is governed by location and tissue-type, but *Vibrio* species are shared among oyster tissues. Front. Microbiol..

[27] Lee MJ, Lee JJ, Chung HY, Choi SH, Kim BS (2016). Analysis of microbiota on abalone (*Haliotis discus hannai*) in South Korea for improved product management. Int. J. Food Microbiol..

[28] Curry KD, Wang Q, Nute MG, Tyshaieva A, Reeves E, Soriano S (2022). Emu: species-level microbial community profiling of full-length 16S rRNA Oxford Nanopore sequencing data. Nat. Methods.

[29] Callahan BJ, Wong J, Heiner C, Oh S, Theriot CM, Gulati AS (2019). High-throughput amplicon sequencing of the full-length 16S rRNA gene with single-nucleotide resolution. Nucleic Acids Res..

[30] Karst SM, Ziels RM, Kirkegaard RH, Sørensen EA, McDonald D, Zhu Q (2021). High-accuracy long-read amplicon sequences using unique molecular identifiers with Nanopore or PacBio sequencing. Nat. Methods.

[31] Wang H, Shankar V, Jiang X (2022). Compositional and functional changes in microbial communities of composts due to the composting-related factors and the presence of listeria monocytogenes. Microbiol. Spectr..

